# Reproducible, Ultra High-Throughput Formation of Multicellular Organization from Single Cell Suspension-Derived Human Embryonic Stem Cell Aggregates

**DOI:** 10.1371/journal.pone.0001565

**Published:** 2008-02-13

**Authors:** Mark D. Ungrin, Chirag Joshi, Andra Nica, Céline Bauwens, Peter W. Zandstra

**Affiliations:** 1 Institute of Biomaterials and Biomedical Engineering, University of Toronto, Toronto, Ontario, Canada; 2 Department of Chemical Engineering and Applied Chemistry, University of Toronto, Toronto, Ontario, Canada; Katholieke Universiteit Leuven, Belgium

## Abstract

**Background:**

Human embryonic stem cells (hESC) should enable novel insights into early human development and provide a renewable source of cells for regenerative medicine. However, because the three-dimensional hESC aggregates [embryoid bodies (hEB)] typically employed to reveal hESC developmental potential are heterogeneous and exhibit disorganized differentiation, progress in hESC technology development has been hindered.

**Methodology/Principal Findings:**

Using a centrifugal forced-aggregation strategy in combination with a novel centrifugal-extraction approach as a foundation, we demonstrated that hESC input composition and inductive environment could be manipulated to form large numbers of well-defined aggregates exhibiting multi-lineage differentiation and substantially improved self-organization from single-cell suspensions. These aggregates exhibited coordinated bi-domain structures including contiguous regions of extraembryonic endoderm- and epiblast-like tissue. A silicon wafer-based microfabrication technology was used to generate surfaces that permit the production of hundreds to thousands of hEB per cm^2^.

**Conclusions/Significance:**

The mechanisms of early human embryogenesis are poorly understood. We report an ultra high throughput (UHTP) approach for generating spatially and temporally synchronised hEB. Aggregates generated in this manner exhibited aspects of peri-implantation tissue-level morphogenesis. These results should advance fundamental studies into early human developmental processes, enable high-throughput screening strategies to identify conditions that specify hESC-derived cells and tissues, and accelerate the pre-clinical evaluation of hESC-derived cells.

## Introduction

Early human embryogenesis is a complex and highly ordered process. Specific genetic programs, activated in response to positional and inter-cellular cues, initiate a cascade of developmental trajectories wherein the progeny of single cells self-organize into tissues and organs. After fertilization, the egg undergoes several rounds of cell division to give rise to the morula. A radial round of cell division then gives rise to the spatially distinct trophectoderm and inner cell mass (ICM). This is followed by the formation of a cavity – the blastocoel – within the structure, now known as a blastocyst, with the ICM at one side. The primitive endoderm then develops at the blastocoelic surface of the ICM, and migrates out across the inner surface of the blastocoel. This then differentiates to the parietal endoderm, which overlies the trophectoderm, and the visceral endoderm (VE), which overlies the ICM. As the VE lays down a basement membrane at the VE-ICM interface, the ICM polarizes and gives rise to a pluripotent epithelium known as the epiblast. This process is depicted in Supplementary Figure S1, both morphologically and as a cellular differentiation hierarchy. The epiblast tissue marks a critical organizational step in the self-assembly of the embryo proper. Subsequently, *via* a co-ordinated multi-cellular migration and differentiation step known as gastrulation, the epiblast gives rise to the three classical embryonic germ layers, ectoderm, endoderm and mesoderm, from which all embryonic tissues derive. The generation of specific functionally-appropriate cell types clustered at particular locations, rather than scattered throughout the developing embryo, is likely a result of both physical (cell adhesion mediated) and biochemical (morphogen-mediated) signals [1]–[3].

The ability to recapitulate aspects of human developmental processes *in vitro* using hESC[4] is of significant interest. From a developmental biology perspective, direct observation and manipulation of the earliest stages of mouse morphogenesis has proved highly informative. In comparison, data on the earliest stages of human development are (not surprisingly) rare and the ability to study reported differences in mouse and human development[5] is expected to be enlightening. From a tissue engineering[6] perspective, appropriate spatial, temporal and mechanical cues are likely to be necessary for differentiation of ESC-derived progenitors capable of generating functional substitutes for damaged or diseased tissue [2], [3], [7], [8]. As it is the processes of development that give rise to these tissues and organs in the first place, these same processes are a logical place to turn in the search for a means of repair or replacement [9]. Unfortunately, application of this paradigm has been hampered by our rudimentary understanding of human embryogenesis – itself a product of the inaccessibility of this stage of development for both practical and ethical reasons. There is thus a need for methods that capture not merely the differentiation of individual stem cells, but the three-dimensional and tissue-level contexts in which this differentiation occurs [10].

In order to recapitulate some of the cues and context inherent to *in vivo* development, many hESC differentiation protocols employ 3-dimensional aggregates known as embryoid bodies (hEB)[11] as an inductive step. While hEB permit the generation of cells arising from all three primary germ layers, in contrast to mouse EB [12]–[16], hEB are commonly derived as a heterogeneous mixture by scraping monolayer cultures to release colonies. This results in hESC aggregate formation and subsequent differentiation which is generally chaotic and disorganized, with wide variability both between and within individual aggregates [11], [17]. This inconsistency imposes significant limitations on the use of hEB both as a model system in which to study human development, and as a source of differentiated cells for applications in both research and clinical settings. *In vivo*, differentiation is not a cell-autonomous process, instead being strongly dependant on the co-evolution of the surrounding tissue [2], [3], [18]–[22], and the ability of the hEB to meaningfully reflect this gestalt for studies of embryogenesis is thus compromised. Similarly, attempts to direct differentiation along specific trajectories *via* macro-environmental variables (i.e. growth factors, medium formulations, etc.) for the purposes of regenerative medicine must compete with a wide range of uncharacterized and potentially contradictory endogenous signals [23]. Consequently, the precision of *in vivo* morphogenesis, where every cell has its place, gives way to differentiation at the level of population averages. The results are inefficiency and a contaminating minefield of residual, potentially tumourigenic and inappropriately differentiated cells [7], [8], [24].

Ideally then, an hEB-based hESC differentiation protocol should have the following characteristics: 1) *reproducibility* – the hEB generated should be consistent in size and shape, within and between experiments; 2) *symmetry* – while current technology does not permit total control over the micro-environment of each individual cell, the production of hEB with maximal symmetry would reduce the *range* of micro-environments present, simplifying interactions with macro-environmental variables that are more easily manipulated and providing a *tabula rasa* for intrinsic organizational processes; 3) *order* – the hEB produced should reflect the tissue-level organization of *in vivo* development (facilitated by point 2); and 4) *ease of use* – the protocol should be able to generate hundreds to thousands of hEB for further study without being unduly labour-intensive. The desire to apply hEB differentiation products in clinical-therapeutic and pharmaceutical-screening contexts adds a fifth target, *scalability* – that is, the processes involved should permit a seamless transition of hEB production across several orders of magnitude, from laboratory observations to practical applications.

We have developed a hEB-generation system which fulfils these requirements; we have used this technology to generate, in a robust and scalable manner, morphologically organized aggregates of hESC capable of multi-lineage differentiation. The tissue-level organization we observe provides independent confirmation of the recently reported non-clonal origin of hESC-derived structures observed in teratomas [10], and will provide a solid foundation for future investigations of the mechanisms of early morphogenesis and development.

## Results and Discussion

### Micro-environment affects cell fate

**Figure 1:**
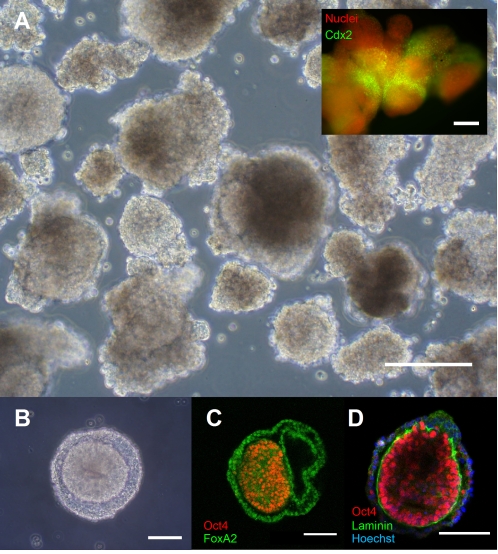
Conventional hESC differentiation protocols result in heterogeneous aggregates with inconsistent organization and structure: A. Conventional differentiating hESC aggregates, formed by scraping colonies of hESC off the culture surface, are predominantly disordered. As hESC colonies differ widely in size and shape, this heterogeneity is passed on to the differentiating aggregate. Consequently the local microenvironment is neither consistent between nor within the aggregates. Scale bar represents 200 microns. *Inset:* Fusing aggregates exhibit expression of the transcription factor CDX2 (green, counterstain 7AAD, red) at points of contact, but not elsewhere, demonstrating the ability of microenvironmental cues to override macro-environmental conditions. Scale bar represents 200 microns. B–D. Rare hESC-derived aggregates exhibit self-organization. Within heterogeneous populations of scraped hESC-derived aggregates, a rare subpopulation of hEB can be observed. These hEB are characterized by the presence of two distinct domains, visible in phase contrast (B). An inner domain is positive for the pluripotency marker Oct4 (C/D – red), while the outer domain is positive for the endodermal marker FoxA2 (shown in green in panel C). A laminin-containing membrane at least partially defines the interface between these two domains (shown in green in panel D, counterstained with Hoechst in blue). Scale bars represent 100 microns.

When generated *via* commonly-employed scraping techniques [11], hEB are extremely heterogeneous in size, shape and organization (Figure 1A). In fact, despite common usage in the hESC field, it is not clear that these objects actually merit the term “embryoid body”, rather than simply “hESC aggregates”. We have thus restricted the use of the term “embryoid body” (EB) to describe aggregates which display clear multi-cellular organization, and use the term “aggregates” or “differentiating aggregates” for the typically-observed hESC-derived structures. This is significant in that no two cells, whether within the same aggregate, or between two aggregates, can be assumed to be experiencing the same microenvironment. As one example, ESC aggregates are prone to adhere to and fuse with one another [25], and we have observed expression of the transcription factor Cdx2 – known to play a significant role in cell fate decisions [26] – in a subset of cells at the junction of fusing hESC aggregates (Figure 1A inset: *green – Cdx2, red – DNA*), demonstrating that incidental differences in micro-environment can override common macro-environmental signals. Micro-environmental heterogeneity might also be expected to interfere with any intrinsic self-organizational capacity of differentiating hESC. Human ESC-derived aggregate heterogeneity thus places significant limitations on their utility as tools to understand development or control hESC differentiation.

### Exploring the capacity of hESC to self-organize towards hEB

Based on the substantial self-organization that pluripotent cells undergo during normal (see Figure S1) and aberrant development, and previous observations on the behaviour of murine and human EB [27]–[29], we hypothesized that the ability to generate tissue-level order is intrinsic to hESC; the reason it is rare in conventional differentiation cultures is that the disorder and irregularity characteristic of the aggregate-generation protocols usually employed leaves only a few aggregates within the homeostatic envelope where this level of organization is accessible. Thus, if a strategy allowing for consistency in aggregate formation could be developed, it should be possible to define conditions which allow these aggregates to self-organize in a robust and predictable manner.

Furthermore, while hESC aggregate formation is commonly employed to induce hESC differentiation, comparatively little attention has been focussed on tissue-level organization within the differentiating aggregates, despite evidence of such potential in murine EBs [30], [31] (a few recent publications have reported the presence of apparent VE at the surface of hEB generated *via* a mechanical dissection technique [29], [32]).

We thus first sought evidence of the capacity of hESC to give rise to tissue-level organization. We employed commonly used differentiation induction techniques (e.g. colony scraping), taking advantage of the heterogeneity of aggregates produced in this manner to broadly survey their capacity for self-organization. A subpopulation (frequency varying between preparations but generally in the range of a few percent of the total) exhibited a multi-laminar radial organization, which was most pronounced and symmetrical among aggregates of two- to three-hundred microns diameter (Figure 1B). Preparations of scraped hESC aggregates were fixed and subjected to immunofluorescent staining, and imaged by fluorescence microscopy (Figure 1C and D). Within the highly-organized subpopulation, distinct segregation into two domains was apparent. An inner domain consisted of cells uniformly positive for the pluripotency marker Oct4, often exhibiting columnar structure with nuclei at the basal surface and formation of an inner cavity. In contrast, cells in the second domain at the aggregate surface were positive for the endodermal marker FoxA2. The interface between the two domains exhibited regions of staining for the basement membrane protein laminin. These results are reminiscent of the epiblast and visceral endoderm domains of the developing embryo (Supplementary Figure S1F), and suggest that hESC are capable of recapitulating at least some aspects of the supra-cellular order that characterizes *in vivo* development. The ability to generate distinct populations of cells with little or no mixing is an important component of the objective of efficient production of clinically useful cell types, uncontaminated by potentially tumourigenic stem cells.

### Forced aggregation of hESC single-cell suspension

Having established that hESC have the inherent capacity to give rise to structured EB, we then proceeded to develop approaches that would allow them to do so more efficiently. We had observed that these organized structures generally formed from more symmetrical aggregates, and within a certain size range, so we sought means of more reliably producing this class of aggregates. More uniform aggregates of mouse ESC are often generated from a single cell suspension using the hanging drop method [12], [13]. However, hESC hanging-drop protocols appear to require small clumps of cells [33], and we wished to minimize the variability and potential for multiple organizing centres that might arise in this situation. The use of cell clumps rather than single cells as inputs would also introduce the possibility of pre-existing organization, and thus complicate future investigations of the process of self-organization within the resulting aggregates. Finally, a single-cell based hEB protocol will simplify studies on the effects of cell mixing on genetic manipulations of hESC differentiation. Building on the hanging-drop concept, mEB have also been generated from single cell suspensions of mESC in microcentrifuge tubes [14], and in 96-well U-bottom microtiter plates [16]. A recently published protocol extended this concept to hESC [34], *via* the addition of a centrifugation step. While this approach provided the benefits of homogenization of the starting population, and consistent cell numbers between replicates, we encountered difficulties in adapting this protocol to our needs. In particular, we found the initial cell pellet formed in a disk configuration, which then collapsed into one or more three-dimensional structures over time, resulting in reduced initial symmetry and more variable aggregate size. We also encountered difficulties in robustly extracting intact aggregates from the wells after formation (see below).

### Improving reliability, symmetry and throughput of aggregate formation

In order to force immediate formation of a more compact pellet, avoid the generation of multiple aggregates from a single well, and increase throughput, we employed 96- and 384-well V-bottom plates. As a metric of success in achieving reliable, consistent and efficient incorporation of hESC into a single aggregate per well, aggregate size (cross-sectional area) was monitored using light microscopy under various conditions. To transition to a true high throughput (HTP) process, we also sought a means of removing the aggregates thus formed from the plates with minimal mechanical disruption, in a manner compatible with automation. We therefore manufactured centrifugation chambers capable of holding a standard-format microtiter plate in an inverted configuration, allowing us to extract the entire contents of several 384-well plates simultaneously. We refer to the aggregates produced in this way as “Spin-In, Spin-Out Embryoid Bodies” (SISO-EB).

**Figure 2:**
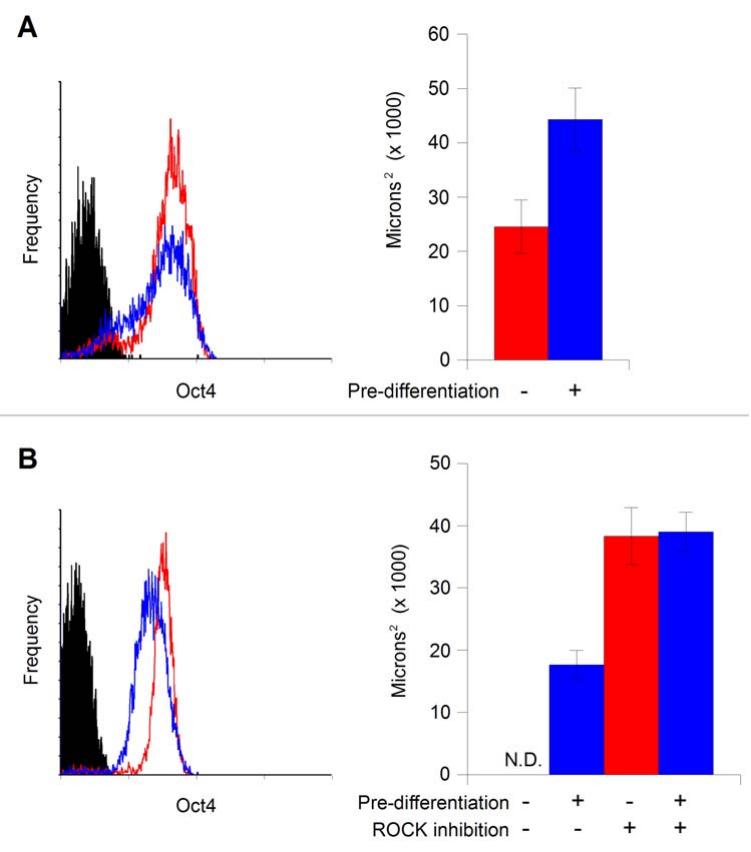
Controlling aggregate formation and stability: A. Pre-differentiation improves aggregate formation and stability. hESC cultured on mouse embryonic fibroblast (MEF) feeders were pre-differentiated with 20% serum for 72 hours prior to aggregate formation, resulting an overall reduction in the population level of Oct4 expression [left panel, red line: standard maintenance culture; blue line: pre-differentiated; black: control (unstained)]. Aggregates formed from 2,000 input cells were substantially larger with treatment (blue bar) than without (red bar). Y axis represents aggregate cross-sectional area in microns^2^, error bars represent one standard deviation. B. The ROCK inhibitor Y-27632 promotes aggregate stability. hESC cells cultured on Matrigel in MEF-conditioned medium with and without pre-differentiation [left panel, red line: standard maintenance culture; blue line: pre-differentiated; black: control (unstained)] were used to form SISO-aggregates in the presence or absence of 10 µM Y-27632. Under these culture conditions, in the absence of both, no aggregates were formed (N.D. - size not determined). With 48 hours pre-differentiation in 20% serum, consistent aggregates were formed (first blue bar). When Y-27632 was added to the suspension of cells without (red bar) or with (second blue bar) pre-differentiation immediately prior to dispensing into the well plate, sizeable aggregates resulted.

We initially observed significant variability in aggregate formation efficacy between experiments, with nominally replicate trials employing the same cell line and number of cells resulting in coherent aggregate formation after 24 hours in one case, and then failing do so in a subsequent attempt (data not shown). After investigating potential causative factors, we noted that population levels of the pluripotency marker Oct4 seemed to affect aggregate cohesion, with aggregates formed from populations of cells with the highest levels of Oct4 exhibiting the least stability. We thus tested whether differentiation induction using serum-containing medium would improve aggregate stability. As shown in Figure 2A, this approach was successful, with 72 hours of serum induction resulting in a substantial increase in aggregate size and symmetry. hEB yield *via* this process approaches 100% (one aggregate per well). The introduction of a spin-out step usable in 384-well format without compromising hEB coherence and reproducibility has resulted in a dramatic increase in the numbers of hEB that can be efficiently generated. In combination with a basic liquid dispensing system, we have been able to easily generate several thousand organized hEB in a single experiment; we expect this HTP approach to be limited only by the available liquid-handling technology. The use of steeper well geometries (96- and 384-well V-bottom plates, vs the 96-well U-bottom plates employed in the original report [34]) also contributes to the direct formation of single, symmetrical aggregates in each well.

We have also noted, anecdotally, that robust aggregate formation can be influenced by other factors such as hESC colony size and distribution on the culture surface, hESC passage number, and protein inductive factors (data not shown). These parameters, which may vary substantially between hESC cultures, even within the same laboratory, may explain the problems we initially encountered in generating “spin-EBs”. In particular, it is interesting that the hESC cultures usually considered to be of highest quality (i.e. uniformly high Oct4 expression) may be less well suited to aggregate formation. Consistent with this we have recently reported that spatial distribution and local cell density-mediated effects influence endogenous and exogenous signalling to regulate hESC propagation [35]. Thus passaging frequency and methodology are expected to affect hESC culture properties in a manner that would impact spin-EB formation, likely a relevant factor in previously published spin-EB protocols [34], [36].

Recently, the p160-Rho associated coiled-coil kinase (ROCK) inhibitor Y-27632 has been reported to promote the survival of hESC after dissociation to single cells, without affecting pluripotency [37]. We therefore tested aggregate formation from 2,000 hESC grown on Matrigel in conditioned medium, with and without 48 hours pre-differentiation and/or exposure to 10 µM Y-27632 during aggregation. Aggregates were spun out the following day, captured on a 40 µm filter, resuspended and imaged. No aggregates were observed in the absence of both pre-differentiation and Y-27632, however either of these alone or in combination resulted in aggregate formation, with the largest aggregates formed in the presence of Y-27632 (Figure 2B). This approach provides a useful alternative to the pre-differentiation technique, and allows investigation into the mechanism-of-action of ROCK inhibition and its use in bioprocesses for generating ESC-derived cells.

### Controlling aggregate size

**Figure 3:**
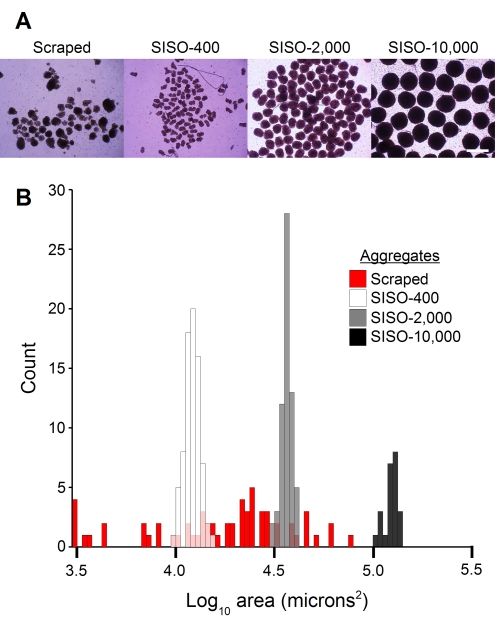
SISO-aggregation allows for the generation of size-controlled aggregates. (A) hEB were generated by scraping, and SISO-aggregates were generated from input populations of 400, 2,000 and 10,000 cells in 384-well plates, and recovered by centrifugation. After imaging in phase-contrast mode, images were thresholded and cross-sectional areas were calculated using the ImageJ software package. Values obtained were extremely consistent, with coefficients of variation of 0.09, 0.06 and 0.08 respectively, *vs* 0.72 for the scraped hEB. (B) The base-10 logarithm of cross sectional area is plotted on a histogram, demonstrating the clear separation between aggregate sizes and dramatic increase in size control over scraping techniques.

Having established a robust protocol for hEB formation in a defined medium formulation, we sought to quantify our ability to regulate aggregate size. Aggregates were formed in 384-well plates from 400, 2,000 and 10,000 cells per well, and spun out after 24 h. Cross-sectional area data was collected *via* phase-contrast microscopy, and quantified using the ImageJ software package. The improvement in aggregate consistency and size control over conventional scraping techniques is striking (Figure 3), with coefficients of variation of less than 0.1 for all three SISO aggregate sizes, compared to >0.7 for the scraped hEB. From previously published work [34] and our own investigations (data not shown), hEB size plays a significant role in establishing differentiation outcomes; thus the ability to control this parameter is important to fundamental and applied applications.

### Micro-textured surfaces for the scalable generation of size-specified organized hEB

While the HTP combination of 384-well plates, automated liquid handling, and a spin-out step allows a significant increase in productivity over the current state-of-the-art, future bioreactor[38] and high-throughput screening (HTS)[39], [40] applications will require further increases in throughput. Successful clinical application of the differentiated progeny of hESC will require methods that bridge the scale gap between the ability to produce thousands or millions of cells at the laboratory bench, and the much larger numbers that will be required *per patient* for clinical protocols (for example a single bone marrow transplant may require hundreds of millions of CD34+ cells [41], while cardiac therapies may require replacement of the billion or more cardiomyocytes lost to an infarct [42]). We thus set out to generate a cost-effective ultra-high throughput (UHTP) method capable of meeting these requirements.

**Figure 4:**
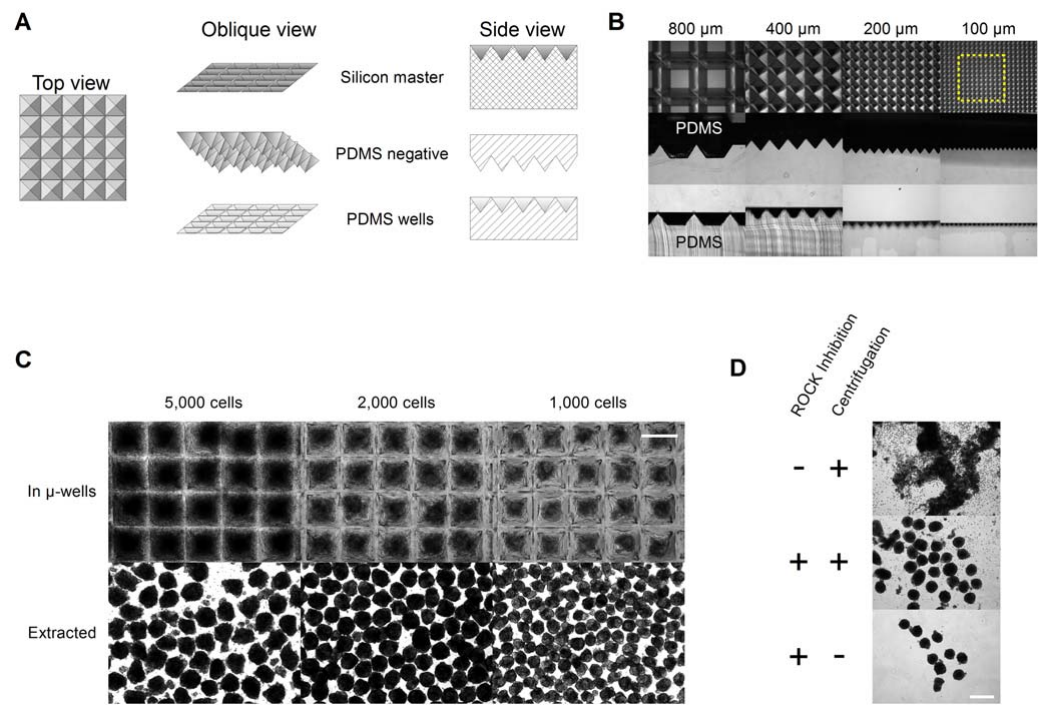
Micropatterned surfaces allow ultra-high-throughput (UHTP) production of size-specified aggregates. Surfaces patterned with arrays of microwells were generated (A) in poly(dimethylsiloxane) *via* serial replica moulding from a pattern etched into a silicon wafer. Imaging of the silicon master, the PDMS negative cast, and the PDMS positive cast show conservation of form across these steps with wells 100, 200, 400 and 800 microns square (B). Note that the 800 micron wells were generated in the form of a truncated pyramid. Dashed yellow square represents one square millimeter. Sections of PDMS textured with 400 micron wells (C) were inserted into individual wells in a 24-well plate. A single-cell suspension of hESC grown on MEF, predifferentiated with serum for 48 hours was dispensed into the well such that each microwell was predicted to capture the desired number of cells. After 24 hours, the well contents were imaged, extracted, and re-imaged. Scale bar represents 400 microns. D. Centrifugation is not required in the presence of ROCK inhibition. Cells grown on Matrigel in defined conditions were dispensed over the microwells in the presence or absence of 10 µM of the ROCK inhibitor Y-27632. Aggregates formed only in the presence of 10 µM of the ROCK inhibitor Y-27632, in the presence or absence of centrifugation. Scale bar represents 400 microns.

To accomplish this goal, we have used a novel approach to generate textured surfaces consisting of numerous collecting volumes, or micro-wells, at the base of a single common liquid volume. Our design parameters included angled collecting surfaces sloped towards a common collecting point, complete surface tiling (to avoid interference and inefficiencies arising from cells landing in the dead space between micro-wells), and biocompatibility. Microfabrication of a master mold, followed by replica molding in poly(dimethylsiloxane) (PDMS) is a technique capable of duplicating extremely fine features [43]. PDMS replica molding has been used to cast arrays of widely spaced vertical-walled micro-wells for ESC culture from templates generated *via* soft-lithographic techniques [44], [45]. Importantly however, soft lithography is not well suited to the generation of the angled collecting surfaces required for this application, and the use of vertical sidewalls also places mechanical-strength limitations on how closely wells can be spaced. We took advantage of the susceptibility of crystalline silicon to anisotropic etching[46] to generate square-pyramidal pits in the surface of a 1-0-0 silicon wafer. As a result of the orientation-specific resistance of internal crystal planes to the etchant, angled sidewalls of near atomic-level perfection parallel to these planes are formed. These sidewalls converge to a point if the etching is allowed to proceed to completion; if the reaction is stopped before completion, a truncated pyramidal well is formed. We then proceeded through two serial rounds of PDMS casting, generating first a negative cast of the silicon wafer, an array of pyramids, and then a positive cast from the negative cast, replicating the array of micro-wells from the original silicon wafer in biocompatible PDMS. Figure 4A depicts a schematic of this process, while panel B contains the corresponding micrographs of the production of 800, 400, 200 and 100 micron square micro-wells (yellow box denotes one square millimetre). The upper row shows a top view of the silicon master, the middle row a section through the PDMS negative casts, and the lower row a section through the final PDMS wells (Figure 4A and B). Note that the 800 micron wells were not etched to completion, demonstrating a truncated pyramidal shape. The apparent rounding of the points of the smaller wells in the lateral sections is an artefact of imperfect alignment between the well point and the plane of the cut.

We next tested the ability of these micro-wells to support hEB formation. hESC cultured on MEF and predifferentiated in serum for 48-hours were dissociated to single cells, and centrifuged onto the micro-textured surfaces. After 24 hours, the micro-wells were imaged, their contents extracted by pipetting, and additional images were acquired. hEB formation and size control are clearly preserved in this high-density format (Figure 4C). We also validated the utility of these micro-wells in the context of ROCK inhibition. For this study we extended our results to hESC cultured on Matrigel in defined, serum-free medium [35], [47]. Of note, coherent aggregates generated under these conditions formed only in the presence of Y-27632. Most significantly, in contrast to the studies performed with pre-differentiated hESC in the absence of Y-27632, centrifugal aggregation was not required for the formation of hEB (Figure 4D). In this study the only xeno- and undefined component in the generation of hEB is the Matrigel upon which the hESC were cultured. ROCK inhibition is similarly effective for hESC cultured on MEF (data not shown).

Our use of micro-textured surfaces allows for an additional several orders-of-magnitude increase in throughput over the gains arising from the SISO-EB protocol. The approximately 100 cm^2^ surface area of a single standard well-plate footprint can accommodate 15,500, 62,500, 250,000 or 1,000,000 micro-wells of 800, 400, 200 or 100 micron size, respectively. As the textured surface is produced *via* replica molding in PDMS at a cost of pennies per gram, much larger total surface areas than this can be practically manufactured. At the same time, tens to thousands of these same micro-wells can be cast into much smaller surfaces, such as a single well in a 96-well plate, for smaller scale investigations, or for high-throughput screening. This potential for direct, effectively unlimited scalability is expected to significantly accelerate clinical and pharmaceutical applications of pluripotent cells by permitting promising basic-research observations to be translated into a production environment rapidly and efficiently, without the need to reformulate processes using a different set of procedures.

Our results combining micro-textured surfaces with ROCK inhibition establish further improvements (Figures 2B and 4D). This approach has an advantage over serum-mediated pre-differentiation in that Y-27632, being a small molecule, is a defined xeno-free reagent. As the input cell suspension is generated in a defined (X-Vivo10^TM^) medium formulation, there is no additional requirement for xenobiotics or undefined components. While we are presently undertaking molecular studies into the effects of ROCK inhibition on hESC fate, published results[37] have not detected effects on hEB lineage specification. Finally, the ability to generate aggregates in this context without the requirement for a centrifugation step renders the process extremely simple, and easily implemented in an automated manner. A laboratory-scale workstation with basic liquid and plate handling capacity would be able to dispense hESC suspensions into microtiter plates, incubate them, and recover the aggregates with no operator intervention.

### Human EB generated using scalable technologies exhibit tissue-level organization

**Figure 5:**
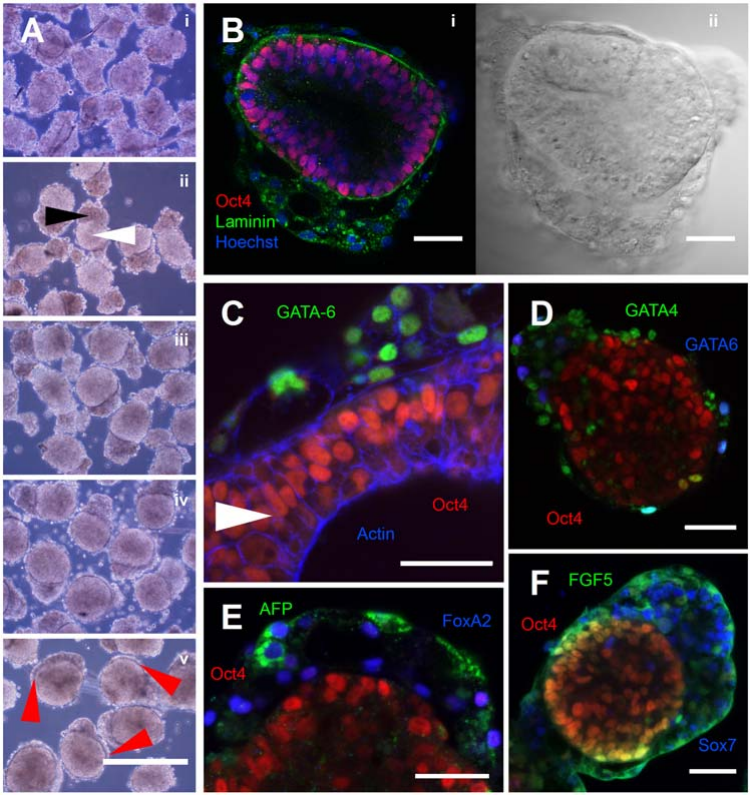
SISO-EB are able to self-organize into highly ordered structures. A. Aggregates formed from 2,000 hESC, imaged immediately after recovery (“day 0”)(Ai), or after 1, 2, 3 or 4 days respectively (Aii-Av). Note self organization into an ordered domain (white arrow) and a disordered domain (black arrow), and progressive encirclement of the ordered domain by the disordered domain over time (red arrows). Scale bar represents 500 microns. B. An aggregate fixed on day 3 of differentiation exhibits Oct4 positive nuclei (red) located basally within the cell over a laminin-containing basement membrane (green) in the ordered domain, with morphologically distinct Oct4 negative cells located outside the membrane in the disordered domain (counterstained with Hoechst, blue). Scale bar represents 50 microns. C. A day 5 aggregate exhibits staining for the endodermal marker GATA-6 (green) in the encircling disordered domain, and the pluripotency marker Oct4 (red) within the ordered domain. Note the tendancy of the Oct4 positive nuclei to align along the interface between the two tissue types, and a tendancy towards columnar morphology (white arrow), both characteristics of epiblast tissue, and the absence of mixing between the two cell types. The actin cytoskeleton, probed with phalloidin, is shown in blue. Scale bar represents 50 microns. D–F. Day 3 aggregates, showing staining for the endodermal markers GATA4, GATA6, AFP and FoxA2, the primitive-endoderm marker Sox7, and FGF5, a marker characteristic of epiblast and overlying VE. Scale bars represent 50 microns.

Having established methodologies to generate size-specified hEB at very high frequencies (∼100%), we proceeded to monitor the structure of aggregates for several days following formation, in order to determine the ability of aggregates formed *via* our protocols to efficiently replicate the self-organization we noted in a rare subpopulation of conventionally-formed aggregate cultures (Figure 1B–D). Aggregates were formed from 2,000 cells, extracted after 24 hours and imaged immediately (day 0) and daily for 4 days. Note the appearance in Figure 5A of two distinct domains in the phase contrast images by day 1 – a highly light-scattering, apparently disordered domain, and a low light-scattering, apparently more ordered domain. The ordered domain exhibits staining for the pluripotency marker Oct4 (Figure 5B and C), while the disordered domain exhibits staining for the endodermal markers GATA-6[48], [49] (Figure 5C and D), GATA4[50] (Figure 5D), AFP[51] and FoxA2[52], [53] (Figure 5E), and the primitive-endoderm marker Sox7[54] (Figure 5F). FGF5, a marker characteristic of epiblast and overlying VE [50], [55], was detected in both (Figure 5F). Indications of epithelial organization are seen in regions where the ordered domain is only a single cell in thickness, with an indication of columnar morphology, and basal localization of nuclei over a laminin-containing basement membrane (Figure 5B and C). These results indicate that SISO-EB self-organize at frequencies approaching unity starting from a hESC culture-derived single-cell suspension. The hEB thus formed recapitulate at least some aspects of early embryogenesis and differentiation, in a manner similar to the previously identified rare EB subpopulation of conventional scraped hESC aggregates (Figure 1B–D) albeit at a much greater efficiency (Figure 5A). Of note, it has recently been reported that organized structures arising from hESC in the context of teratomas are non-clonal in origin – that is, order emerges *via* the coalescence of multiple cells, rather than being inherited from a single progenitor [10]. In the context of our single-cell-suspension-derived hEB, the rapidity with which these structures form precludes the possibility that they are clonally derived, providing independent confirmation of this observation in an alternative system, and further emphasizing the importance of micro-environment in determining differentiation trajectories.

The appearance of sharply demarcated spatial domains consisting of distinct cell types within our hEB also represents a significant advance. Whereas conventional hESC differentiation protocols are able to produce various cell types, they have not been successful in generating the tissue-level order and purity present in the structures we have generated. Given that *in vivo*, the pluripotent epiblast gives rise to the three canonical germ layers *via* organized tissue-level movements, it is reasonable to expect that further refinements to this protocol might render possible the organized and directed tissue-level differentiation of more specified tissue types. Such progress may be a necessary step towards *in vitro* organogenesis.

### hEB generated via our forced-aggregation techniques can differentiate to cell types of clinical and scientific interest

**Figure 6:**
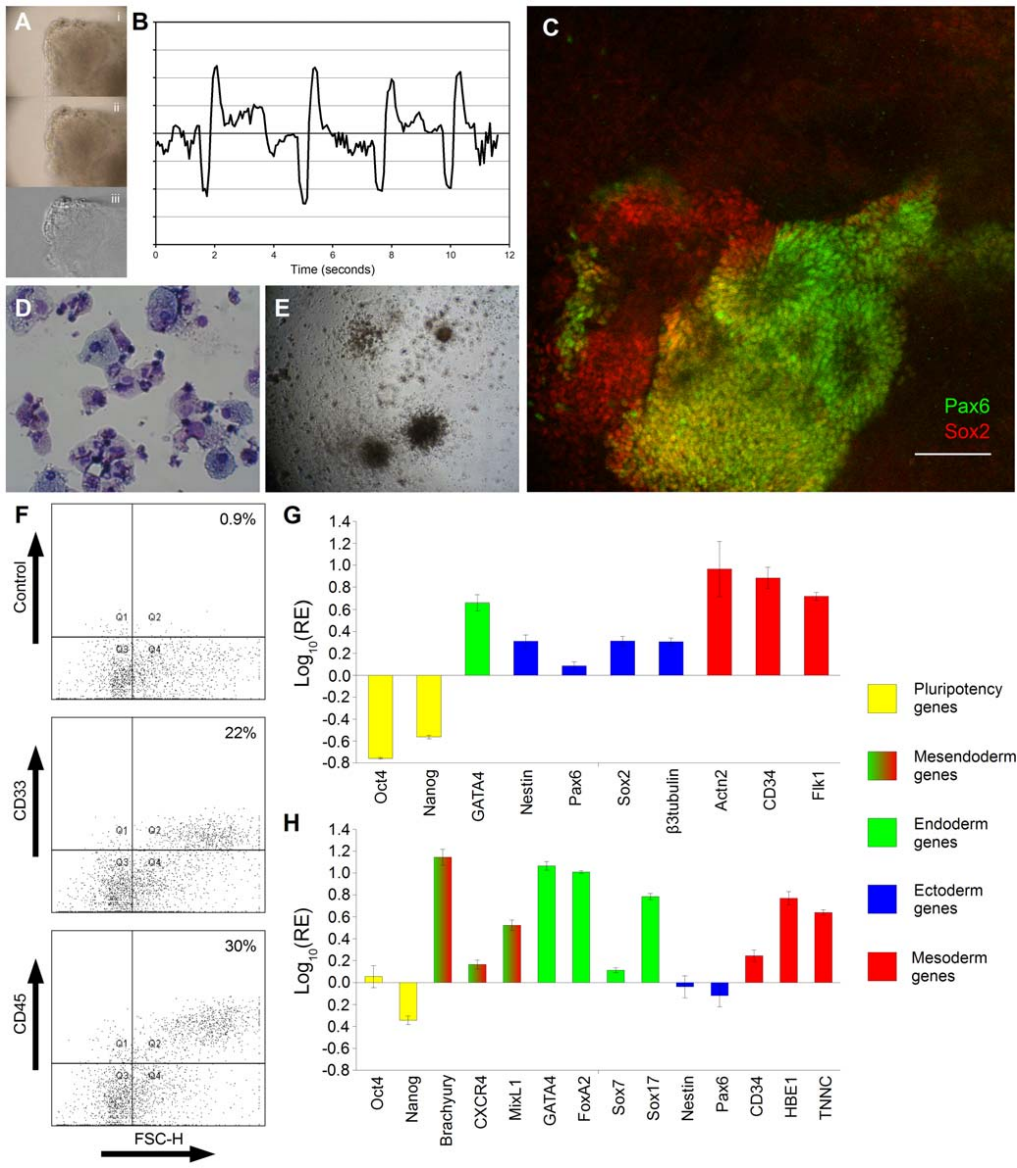
SISO-EB are able to give rise to cardiac, hematopoetic and neural cells. Cardiac differentiation from hEB differentiated for 12 days in suspension culture in serum-containing medium. A) two frames from a video recording of a contractile aggregate, shown before (Ai) and during (Aii) a contraction. Panel Aiii is derived by subtracting panel Ai from panel Aii. The contraction trace (B) was generated by integrating the subtraction image derived from successive frames in the video over the area of contraction, and plotting a 5-point moving average. Neural rosettes (C) were observed after 11 days in culture (4 days in suspension followed by 7 days adherent), staining positive for Pax6 (shown in green) and Sox2 (shown in red). Hematopoetic differentiation was observed after 28 days using Cytospin (D), CFC (E) and flow cytometric (F) assays. G. Quantitative RT-PCR results from aggregates formed from 10,000 pre-differentiated cells in the absence of ROCK inhibition, differentiated for 4 days in suspension followed by an additional 3 days in adherent culture shows down-regulation of pluripotency genes, and up-regulation of markers for endodermal, ectodermal and mesodermal lineages. H. Quantitative RT-PCR results from aggregates formed from 4,000 untreated cells in the presence of ROCK inhibition, differentiated for 4 days in suspension, showed upregulation of mesodermal and endodermal markers, including markers for mesendodermal precursors, but only limited upregulation of the primitive endoderm marker Sox7.

To ensure that our new protocols did not interfere with hESC aggregate developmental potential, we next verified that our methodology could be used to generate the specific differentiated lineages typically derived from differentiated hESC aggregates. Differentiated SISO-EB gave rise to beating cardiomyocytes (Figure 6A and B), rosette-forming neural cells (Figure 6C), and CD33^+^ and CD45^+^ cells and their associated blood-lineage colony-forming cells (CFC) (Figure 6D–F). Quantitative RT-PCR analysis of hEB formed from 10,000 pre-differentiated cells in the absence of ROCK inhibition, differentiated for 4 days in suspension followed by 3 days adherent culture, showed upregulation of markers of endodermal, ectodermal and mesodermal lineages (Figure 6G) and concomitant reductions (relative to input cells) in the expression of the pluripotency-associated markers Oct4 and Nanog. In a complementary experiment, hEB formed from 4,000 untreated cells in the presence of ROCK inhibition, differentiated for 4 days in suspension, showed upregulation of mesodermal and endodermal markers (Figure 6H). While only a slight increase in levels of Sox7, a marker characteristic of primitive endoderm [54], was observed, much larger increases in other endodermal markers (GATA4, FoxA2 and Sox17) were seen, suggesting the presence of definitive endoderm. This is supported by increased levels of early mesendoderm markers Brachyury and MixL1 indicating the presence of precursor cell types.

The distinct differentiation trajectories observed under these two conditions illustrate the potential for manipulation of output cell populations by controlling the input population and/or the presence of exogenous factors and interactions during hEB-mediated differentiation. While the “default” (endogenous) differentiation programs induce multiple lineages including extraembryonic endoderm, by overriding endogenous signalling with exogenous cytokines and environments [56], [57], we expect to be able to generate relatively pure populations of various cell types. In preliminary experiments, we have observed that sequential addition of specific combinations of cytokines to hEB produced in the microwell device can consistently give over 80% purity of a desired cell type (data not shown). Given the substantially improved homogeneity of hEB produced using our technology, this approach should allow for more effective and higher throughput production of differentiated cells than previously possible.

### Summary

We present here methods for the production of hEB that fulfil the criteria of *reliability*, *symmetry*, *order*, *ease-of-use* and *scalability.* Our approach is well suited for substitution wherever more conventional, less controlled aggregate-based hESC differentiation induction protocols are currently employed. This technology is also broadly applicable to aggregation of other cell types, including pluripotent lines produced via recently reported iPS techniques [58], [59]. The single-cell suspension step integral to this protocol is ideal for mixing experiments, and will also facilitate genetic manipulation of the hESC. In addition to the common extant applications of hEB, aggregates generated *via* these methods exhibit consistent levels of symmetry and self-organization not attainable using more traditional methods, laying the foundations for a new paradigm of *in vitro* tissue-level differentiation. Finally, the dramatic increase in hEB output achieved using these methods now permits hEB-mediated differentiation applications not previously attainable.

## Materials and Methods

### Cell culture methods

Unless otherwise specified, hESC were cultured on an irradiated mouse embryonic fibroblast (MEF) feeder layer as described elsewhere [4], in KO-DMEM (Invitrogen, cat# 10829-018), 20% KOSR (Invitrogen, cat# 10828-028,), 100 µM non-essential amino acids (Invitrogen, cat# 11140-050), 2 mM Glutamax (Invitrogen, cat# 35050-061), 100 µM β-mercaptoethanol (Sigma-Aldrich, cat# M7522) with 4 ng/mL FGF2 (Peprotech, cat# 100-18B). In some cases, hESC were cultured on Matrigel (BD Biosciences, cat# 356231) coated (1∶30 dilution) plasticware in either MEF-conditioned medium with 12 ng/mL FGF2 (Peprotech, cat# 100-18B)[60] or X-Vivo10™ medium (BioWhittaker cat# 04-380Q) with 20 ng/mL FGF2 (Peprotech, cat# 100-18B), 5 ng/mL Activin A (R&D cat# 338-AC-025), and 0.1 ng/mL TGFβ (R&D cat# 240-B-010) [35], [47]. In all cases, efforts were made to ensure even seeding of hESC across the well, relatively uniform colony size, and an absence of large clumps of cells. Experiments presented here were carried out in the H9 cell line; however the effectiveness of these protocols for hESC aggregate formation was also confirmed in the CA-1 and I6 cell lines. Rare organized hEB were first observed in the CA-1, CA-2 and H9 cell lines.

### hEB formation by scraping

The method used to generate standard hEBs was based on published protocols [61]. Briefly, hESC colonies were incubated with 0.1% Collagenase IV (Invitrogen, cat# 17104-019) at 37°C for 10 minutes. The Collagenase IV was removed and replaced with the desired medium formulation. hESC colonies were scraped from the tissue culture plastic using a cell scraper (Sarstedt, cat# 831830), and the resulting suspension gently pipetted to generate a distribution of clumps of the desired size. These clumps were then transferred to an ultra-low attachment non-tissue culture treated plate (Corning, cat# 3471) and cultured in suspension.

### Flow cytometry for Oct4

Flow cytometric analysis for Oct4 expression was carried out as described elsewhere [62]. In brief, hESC were fixed and permeabilized with an Intraprep Permeabilization Kit (Beckman Coulter, cat# IM2389), and sequentially probed with primary antibody to Oct3/4 (BD Transduction Laboratories, cat# 611202) and FITC conjugated secondary antibody (Sigma-Aldrich, cat# F-2772), and assayed on a Beckman Coulter Epics XL flow cytometer.

### hESC preparation for aggregate formation

Prior to preparation of single-cell suspension, hESC were pre-differentiated when specified by replacing the normal culture medium with differentiation medium (DM – consisting of DMEM, 20% FCS, 1% NEAA, 1% beta-mercaptoethanol, 1% L-glutamine, 0.5% Pen/Strep) for the specified time period (generally 48 or 72 hours) prior to harvest. Cells were dissociated with TrypLE™ (Invitrogen, cat# 12605-028) for 3–10 minutes at 37°C, and optionally passed through a 40 micron filter unit. After centrifugation, cells were resuspended at the desired concentration in X-Vivo10™ defined medium (BioWhittaker cat# 04-380Q).

### Plate preparation for aggregate formation

96- or 384-well microtiter plates (Costar, cat# CS003896, Whatman, cat# 7701-5101, lids cat# 7704-1001) were loaded with 40 or 25 µL per well respectively of 5% (w/v) Pluronic F-127 (Sigma-Aldrich, cat# P2443) in PBS (Gibco, cat# 14190), and incubated for at least 30 minutes at room temperature. Wells were then emptied immediately prior to use.

### Spin-in spin-out aggregate formation

Cells were loaded into plates prepared as above in 100 or 25 µL volume for 96- and 384-well plates respectively. Plates were then centrifuged for 5 minutes at 200×g, and then incubated overnight at 37°C, 5% CO_2_. The following day, well contents were recovered either *via* inverted centrifugation in custom machined plate carriers for 1 minute at 50×g, or pipetting with large bore “genomic” pipette tips (Molecular Bioproducts, cat# 3531). The resulting aggregates were then optionally dispensed over an inverted 40 micron filter unit to eliminate unincorporated cells and debris, and then washed off and recovered. Aggregate size was assessed from calibrated photomicrographs using the Analyze Particles function of the WCIF ImageJ software package (http://rsb.info.nih.gov/ij/ and http://www.uhnresearch.ca/facilities/wcif/fdownload.html).

### hEB differentiation

hESC aggregates were cultured in suspension for 4 days in hESC differentiation medium, containing KO-DMEM (Invitrogen, cat# 10829-018), 20% FBS (Gibco 12483-020), 100 µM non-essential amino acids (Invitrogen, cat# 11140-050), 2 mM Glutamax™ (Invitrogen, cat# 35050-061), 100 µM β-mercaptoethanol (Sigma-Aldrich, cat# M7522), 1 U/mL penicillin and 1 µg/mL streptomycin (Invitrogen, cat# 15140-122), followed by further suspension culture or by plating onto 0.5% gelatine coated tissue culture treated dishes, and culture as EB outgrowths for the remainder of differentiation culture. Cardiac differentiation was detected as coordinated beating. Neural differentiation was detected *via* the formation of neural rosettes.

For hematopoetic differentiation, hEB were cultured for 14 days in differentiation medium with cytokines as described elsewhere [63]. hEBs were then dissociated into single cells by trypsinization (0.1% trypsin-EDTA) and filtration through 40 µm mesh to obtain a uniform single cell suspension. 100,000 cells in 200 µl differentiation medium were added to 2 ml methyl cellulose medium supplemented with hematopoietic growth factors (Stem Cell Technologies Inc., cat# H4435), plated in 30 mm petri dishes and incubated for 2 weeks and scored for CFC using established criteria. For cytospin preparation, the hematopoietic colonies were extracted from methocult cultures and Wright-Giemsa staining of cytospin preparations was carried out to assess nuclear and cytoplasmic morphology of the cells. Surface immunofluorescence of cells extracted from colonies in methylcellulose was assessed by staining with anti-CD33 and anti-CD45 antibodies directly conjugated with Phycoerythrin after adjusting for non-specific antibody binding with appropriate isotype control (Immunotech, Cat# IM1179 (CD33-PE), IM2078 (CD45-PE) and IM0670 (IgG1-PE)). Data was acquired on FACS Canto (Becton-Dickinson) and analysed with DIVA software.

### Quantitative RT-PCR

Total RNA was extracted from the cells by homogenization in Trizol Reagent (Invitrogen cat# 15596-026) and extraction with chloroform followed by precipitation with iso-propyl alcohol as per the manufacturer's instructions. The RNA was then purified with RNeasy columns (Qiagen, cat# 74104) with an on-column DNaseI digestion step, as per the manufacturer's instructions. Purified RNA was used to generate cDNA using Superscript-III reverse transcriptase (Invitrogen, cat# 18080-093) as per the manufacturer's instructions. 10 ng of cDNA was used per PCR reaction using iQ-SYBR-green master mix (BioRad, cat# 170-8882) or Power SYBR Green PCR Master Mix (Applied Biosystems, cat# 4368702) in triplicate. Relative expression of various marker genes was determined by delta-delta Ct method with Pfaffl modification[64] and with the expression of beta-actin as internal housekeeping reference. The primer sequences are listed in Supplementary Table S1 and were derived from the MGH Primerbank [65]. Specificity of amplification was assured with analysis of dissociation curves of all reactions. PCR was also performed with “no reverse transcriptase” control for all samples and no amplification was detected in these controls.

### Immunofluorescence microscopy

hEB were suspended in 50uL of HF (Hank's Balanced Salt Solution [Invitrogen, cat# 14175-103]+2% FBS) in an microcentrifuge tube, to which 100 µL of Intraprep Reagent 1 (Beckman Coulter, cat# IM2389 ) was added, and incubated on a rocker table at 37°C overnight. The next day they were allowed to settle, and washed 5× on a rocker table with 1.5 mL of HF+4%BSA, changing wash every hour. They were then resuspended in 100uL of Intraprep Reagent 2 (Beckman Coulter, cat# IM2389) and 5 uL of 4%BSA, in the presence of the desired primary antibody, and incubated on a rocker table at 37°C overnight. They were then again washed 5× on a rocker table with 1.5 mL of HF+4%BSA, changing wash every hour, and resuspended in 100 uL HF with 5 µL of 4%BSA in the presence of the desired secondary antibody conjugated to one of Alexa-488, -546, or -647 (Invitrogen), with Hoechst 33342 dye (Sigma-Aldrich, cat# B2261) or Alexa-350 conjugated phalloidin (Invitrogen, cat# A2281) if desired. After incubating on a rocker table at 37°C overnight they were again washed 5× on a rocker table with 1.5 mL of HF+4%BSA, changing wash every hour. For imaging, the hEB were then dispensed onto a microscope slide suspended in HF, inside a 1 mm silicone rubber gasket (Sigma-Aldrich, cat# S6560), and covered with a coverslip. They were then imaged through the coverslip using epifluorescence or laser-scanning confocal microscopy. Adherent cells were fixed in 3.7% formaldehyde for 10 minutes at 37°C, washed 3× in PBS, permeabilized with 100% methanol for 3 minutes at room temperature, washed 3× in PBS, and blocked overnight at 4°C in PBS with 10% FBS. Primary antibody was added in 10% FBS in PBS, and incubated overnight at 4°C. Cells were then washed 3× in PBS, and secondary antibody was added in PBS with 10% FBS. They were then incubated for 2 hours at room temperature, washed 3× in PBS, and imaged in PBS.

### Generation of micro-textured surfaces

Silicon master molds were generated by the Canadian Microsystems Consortium (http://www.cmc.ca/). Briefly, wafers of 1-0-0 crystalline silicon were coated with a silicon nitride layer, which was then selectively removed where micro-wells were to be formed, and the wafer was then anisotropically etched to generate arrays of square-pyramidal pits. After subsequent removal of the remaining silicon nitride layer, the wafer was used as a template for replica molding in poly(dimethylsiloxane). The wafer was washed with a 5% solution of Pluronic-F127 (Sigma-Aldrich, cat# P2443) in water to act as a release agent, drained, and allowed to air dry. Sylgard-184 (Dow Corning) was combined with the curing agent and de-gassed as per the manufacturer's instructions, and then poured over the wafer and allowed to cure for 3 hours at 70°C, or overnight at 37°C to generate the negative cast. This process was then repeated to using the negative cast as a template, to generate the positive cast containing the micro-wells.

## Supporting Information

Figure S1 Early embryogenesis is a highly ordered process. After fertilization, the egg (A) undergoes several rounds of cell division to give rise to the morula (B). A radial round of cell division then gives rise to (C) the spatially separate trophectoderm and inner cell mass (ICM). This is followed by (D) the formation of a cavity - the blastocoel - within the structure, now known as a blastocyst. The (E) primitive endoderm then develops at the blastocoelic surface of the ICM, and migrates out across the inner surface of the blastocoel. This then differentiates to the parietal endoderm, where it overlies the trophectoderm, and the visceral endoderm (VE), where it overlies the ICM. As the VE lays down a basement membrane at the VE-ICM interface, the ICM polarizes and gives rise to a pluripotent epithelium known as the epiblast (F). This same process is also depicted as a cell differentiation hierarchy (G), where the epiblast then goes on to give rise to the entire developing organism.
(0.27 MB TIF)

Table S1 Quantitative RT-PCR primer sequences
(0.08 MB DOC)
