# Supplementary material for: Reproducible, Ultra High-Throughput Formation of Multicellular Organization from Single Cell Suspension-Derived Human Embryonic Stem Cell Aggregates
Source: PLoS One. 2008 Feb 13;3(2):e1565. doi: 10.1371/journal.pone.0001565 (PMC2215775; doi:10.1371/journal.pone.0001565)
Supplement: Supplementary file 1 [file pone.0001565.s002.doc]

Gene	5' Primer	3' Primer
beta-Actin	CATGTACGTTGCTATCCAGGC	CTCCTTAATGTCACGCACGAT
Oct4	CTTGAATCCCGAATGGGAAAGGG	CCTTCCCAAATAGAACCCCCA
Nanog	CAAAGGCAAACAACCCACTT	TCTGCTGGAGGCTGAGGTAT
GATA4	ATGTCCCGGCGAAAGCAAA	GCTCAGATTTTCAGGCCCAAA
Nestin	GGCGCACCTCAAGATGTCC	CTTGGGGTCCTGAAAGCTG
Pax6	ATGTGTGAGTAAAATTCTGGGCA	GCTTACAACTTCTGGAGTCGCTA
Sox2	GCCGAGTGGAAACTTTTGTCG	GCAGCGTGTACTTATCCTTCTT
B3tubulin	CGGTGGTGGAACCCTACAAC	AGGTGGTGACTCCGCTCAT
Actn2	GGGTCCGTTTGCCAGTCAG	GGCTTTCCTTAGGTGGGAGTT
CD34	GCGCTTTGCTTGCTGAGTTT	GCCATGTTGAGACACAGGGT
Flk1	ACTTTGGAAGACAGAACCAAATTATCTC	TGGGCACCATTCCACCA
Brachyury	TGCTTCCCTGAGACCCAGTT	GATCACTTCTTTCCTTTGCATCAAG
CXCR4	CACCGCATCTGGAGAACCA	GCCCATTTCCTCGGTGTAGTT
MixL1	CCGAGTCCAGGATCCAGGTA	CTCTGACGCCGAGACTTGG
FoxA2	GCGACCCCAAGACCTACAG	GGTTCTGCCGGTAGAAGGG
Sox7	ACGCCGAGCTCAGCAAGAT	TCCACGTACGGCCTCTTCTG
Sox17	CCTGGGTTTTTGTTGTTGCT	GCTGTTTTGGGACACATTCA
HBE1	TGCATGTGGATCCTGAGAAC	CGACAGCAGACACCAGCTT
TNNC	ACGACAGCAAAGGGAAATCTG	TGTTCTTGTCTCCGTCCTTCA
